# Brazilian Scale for Evaluation of Mental Health Care Needs: Additional Evidence

**DOI:** 10.11606/s1518-8787.2023057005347

**Published:** 2024-04-01

**Authors:** Joana Moscoso Teixeira de Mendonça, Flavio Rebustini, Ana Alice Freire de Sousa, Ilana Eshriqui, Daiana Bonfim, Leticia Yamawaka de Almeida

**Affiliations:** I Hospital Israelita Albert Einstein Centro de Estudos, Pesquisa e Prática em Atenção Primária à Saúde e Redes São Paulo SP Brasil Hospital Israelita Albert Einstein. Centro de Estudos, Pesquisa e Prática em Atenção Primária à Saúde e Redes (CEPPAR). São Paulo, SP, Brasil; II Universidade de São Paulo Escola de Artes, Ciências e Humanidades Departamento de Gerontologia São Paulo SP Brasil Universidade de São Paulo. Escola de Artes, Ciências e Humanidades. Departamento de Gerontologia. São Paulo, SP, Brasil

**Keywords:** Mental Health, Primary Health Care, Population Health Management

## Abstract

**OBJECTIVE:**

To investigate validity evidence of the Brazilian Scale for Evaluation of Mental Health Care Needs (CuidaSM).

**METHODS:**

This is a psychometric study, which seeks additional evidence of internal structure. Data collection was carried out in 11 Primary Health Care (PHC) services , which implement the Health Care Planning (HCP) methodology, distributed across the five Brazilian regions. The preliminary version of CuidaSM, containing a block self-referred by the user and another block evaluated by PHC professionals, was applied to users aged 18 or over who attended the PHC services for consultation with a higher education professional. The techniques of confirmatory factor analysis and network analysis were used to investigate validity evidence. For the primary data of the confirmatory factor analysis, the factorial loads and the item’s predictive power (R2) were used. Six model adjustment indices were adopted and reliability was measured by three indicators using Bayesian estimation.

**RESULTS:**

A total of 879 users participated in the study. By confirmatory factor analysis, factorial loads ranged from 0.43 to 0.99 and R2 from 0.19 to 0.98. Both the primary indicators and the model adequacy indices were established at satisfactory and consistent levels. The network analysis showed that the items were appropriately associated with their peers, respecting the established dimensions, which again indicates the sustainability and stability of the proposed model.

**CONCLUSIONS:**

The study findings confirm a consistent and reliable model of the instrument, through a combination of techniques. Considering the importance of using solid instruments in clinical practice, CuidaSM is a promising tool for population-based management and network care organization, aligned with HCP proposals.

## INTRODUCTION

Health Care Planning (HCP) is a methodology that seeks to provide health professionals with the development of skills aimed at planning and organizing health care, considering the needs of users in their territory. In general terms, the proposal aims to integrate the Health Care Network, using strategies to improve the organization of macro and micro processes of primary health care (PHC) and specialized outpatient care ^1^.

In the context of PHC, the metaphor of building a house is used to illustrate the theoretical operational model of its social construction, in which the basic macro and micro processes constitute the foundation and provide support for meeting the demands of the target population^1^. From this perspective, the risk stratification of chronic conditions, based on the Chronic Conditions Care Model , emerges as a fundamental element for planning and implementing actions in services, in accordance with population-based management^1,2^.

Notably, the conduction of this process, by enabling the identification of users’ needs and differentiating them by groups/strata, directs the professional’s decision-making in relation to the type of care, resources and technologies specific to clinical management^2^ and organization of assistance in different lines of care.

In the context of mental health (MH) care, in the face of a global scenario of scarce resources and the therapeutic gap for mental disorders^3,4^, PHC has emerged as a strategic component for care^5,6^. However, the use of tools to quantify and organize this demand for services has been considered a challenge among professionals^7^.

The MH care lines developed by the Ministry of Health^8^recommend the use of instruments that, in general, aim to facilitate the detection of specific conditions, such as depression, anxiety, and problems related to alcohol use. However, there is a lack of instruments that support professionals in stepped care model sin the territory.

In this sense, the Brazilian Scale for Evaluation of Mental Health Care Needs (known as CuidaSM)^9^was developed, aligned with the concept of population-based management, to facilitate the recognition of specific needs for mental health care, configuring itself as a potential tool to collaborate with PHC teams in operationalizing the basic macro-process of stratification and, consequently, in the provision and organization of care.

A previous study demonstrated evidence of validity of CuidaSM, via exploratory factor analysis, thus, this instrument is composed of 31 dichotomous items, covering five dimensions in a self-referred block (Social Relationship, Functionality, Autonomy, Impulsiveness and Aggressiveness, Spirituality) and three dimensions in a block applied by a higher-level PHC professional (Violence, Self-aggression and Suicidal Behavior, Care Plan)^9^. From its application, it is possible to classify people who use PHC into four strata of mental health care needs (MHCN) (low, moderate, high, and very high)^9^. In this context, CuidaSM supports the process of stepped care based on objective data on MHCN. Furthermore, it has the potential to contribute to effective communication between different points of care in the network, with decision-making regarding the right therapy, at the right time, for the right user, and, therefore, with the more rational use of technical and human resources, assisting in care programming.

Considering that to date there are no other validated instruments to measure MHCN in the Brazilian population, as well as the relevance of using, in clinical practice, solid instruments in terms of psychometric properties and in line with current recommendations regarding multiple tests to adjust an instrument^10^, this study aimed to investigate evidence of the validity of CuidaSM.

## METHODS

### Design

This is a study of additional evidence of the internal structure of CuidaSM, carried out through confirmatory factor analysis (CFA) and network analysis, according to the current recommendation of the American Educational Research Association, American Psychological Association and the National Council on Measurement in Education^10^. The study was approved by the Research Ethics Committee of Hospital Israelita Albert Einstein (CAAE: 12395919.0.0000.0071).

### Data collection

The preliminary version of CuidaSM, consisting of 43 items (23 in a self-referred user block and 20 in a clinical assessment block carried out by a PHC professional), as described in a previous study^9^, was applied to users registered in 11 PHC services, totaling 27 family health teams. Collection took place between November 2021 and August 2022, and was conducted by a team of previously trained researchers.

The PHC services that made up the research field were defined based on their location, in municipalities that use the HCP methodology and the inclusion of at least one service in each geographic region of the country, including: one PHC service in the North region (Roraima); one PHC service in the Northeast region (Pernambuco); two PHC service in the Midwest region (Mato Grosso); five PHC service in the Southeast region (three in São Paulo and two in Minas Gerais); and two PHC service in the South region (Paraná).

After contacting the service management to agree on the activity schedule, the responsible researcher held virtual meetings with the units’ teams to present the study proposal. Furthermore, in these meetings, doubts were clarified and data collection was prepared, which included health professionals for the clinical block evaluation.

The operationalization of data collection in the PHC services was carried out in two moments. Initially, users were approached by researchers in the PHC services waiting rooms and invited to participate in the research, by reading and expressing acceptance of the Informed Consent Form. At this time, they answered a questionnaire composed of sociodemographic items, clinical profile, and the self-referred block of the CuidaSM scale.

Subsequently, during the user’s consultation with the PHC health professional, the clinical assessment of the CuidaSM scale was carried out. For this moment, a printed version of the instrument was used, which was collected by the researchers at the end of the day. Data recording was carried out in a tablet, through Research Electronic Data Capture (REDCap^©^).

The eligibility criteria for users were: being 18 years old or over and being at the PHC service for care in individual consultation with a doctor, a nurse or health professionals from the multidisciplinary team. Those who attended the service for emergency or dental care or procedures (such as vaccination, dressing and medication) or who did not complete the “health professional evaluation” block were excluded.

### Data analysis

#### Confirmatory Factor Analysis (CFA)

For the primary data of the CFA, the factorial loads and the predictive power of the item (R2) were used^2^. The model adjustment indices adopted were: χ^2^/df; Non- Normed Fit Index (NNFI ≥ 0.95); Comparative Fit Index (CFI ≥ 0.95); Goodness Fit Index (GFI ≥ 0.95); Root Mean Square Error of Approximation (RMSEA < 0.08) and Root Mean Square of Residuals (RMSR ≥ 0.8). The model tested in the EFA was the factorial solution found in the initial study^9^of the exploratory factor analysis.

Reliability was measured by three indicators: Cronbach’s alpha^14^; Greatest Lower Bound (GLB)^15^; and Omega^16^, all of the three using Bayesian estimation^17^.

#### Network Analysis

In the last decade there has been an extension of the application of network analysis to different scenarios and applications, such as: symptom assessment^18^, psychological networks and psychopathologies^19,20^, post-traumatic stress^21,22^, schizophrenia^23^, anxiety^24^and instrument development measuring^25^. Nevertheless, in Brazil, studies using network analysis are rare^30,31^.

It is important to understand how it can be useful in the search for validity evidence. According to Newman^32^(2010), the analysis is composed of two stages: in the first, it estimates a statistical data model, from which some parameters can be represented as a weighted network between the observed variables; and in the second stage, the structure of the weighted network is analyzed, using measures taken from graph theory.

This study used the High-dimensional Undirected Graph Estimation (Huge)^33^and Extended Bayesian Information Criterion (Ebic) as criterion. Huge works with two estimation procedures: 1) the neighborhood search algorithm^34^and 2) the Lasso graph algorithm^35^. The graph nodes were positioned using the Fruchterman and Reingold algorithm^36^, which is based on the strength and connectivity between the nodes. Each node represents an instrument item.

To evaluate the generated model, four indicators were adopted: Betweenness, which evaluates the efficiency with which a node connects to others; closeness, which evaluates the ease with which information reaches other nodes from a specific node; strength (or degree), which represents how much a node is connected to the rest of the network^37^; and, finally, expected influence, which aims to assess the nature and strength of the cumulative influence of a node within the network^23^and, therefore, the role it can be expected to play in its activation, persistence and remission^38^.

For both techniques, a bootstrap of 5,000 was applied. Analyses were performed using JASP 16.04.

## RESULTS

1,219 people agreed to participate in the study, of which 879 had both CuidaSM blocks completed and were included in this study. Among these, the mean age was 45 years (SD = 16.7). The majority were female (74.3%), married or in a common-law marriage (46.5%), not beneficiaries of social programs (76.0%) and with nine or more years of schooling (54.2%). The participants lived mainly in the Southeast region (58.4%), followed by the South (11.1%), Midwest (11.0%), North (5.4%), and Northeast (4%).

**Figure 1:**
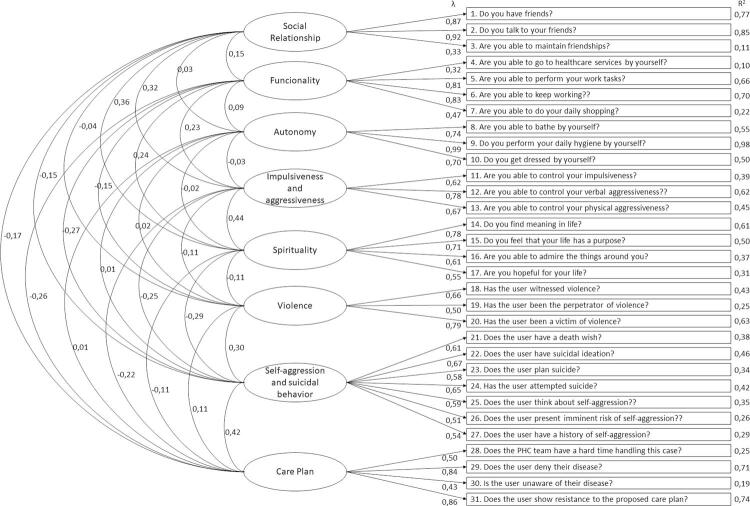
Pathways diagram.

Using exploratory factor analysis, factorial loads ranged from 0.43 to 0.99, with standard errors ranging from 0.004 to 0.018 and the item’s predictive capacity (R2) from 0.19 to 0.98 (Figure 1). All at satisfactory levels. In addition to the primary indicators, the model fit indices were established at: X2/df (245) = 1.64, p = 0.001; NNFI = 0.97, CFI = 0.98, GFI = 0.98, RMSEA = 0.06, and RMSR = 0.03.

The reliability indices with Bayesian estimation were: Cronbach’s alpha = 0.81 (95%CI 0.79–0.82), McDonald’s omega = 0.78 (95%CI 0.76–0.81) and GLB = 0.93 (95%CI 0.92–0.93). All indicators at appropriate levels. In this way, both the primary indicators and the model adequacy indices were established at satisfactory and consistent levels.

**Figure 2:**
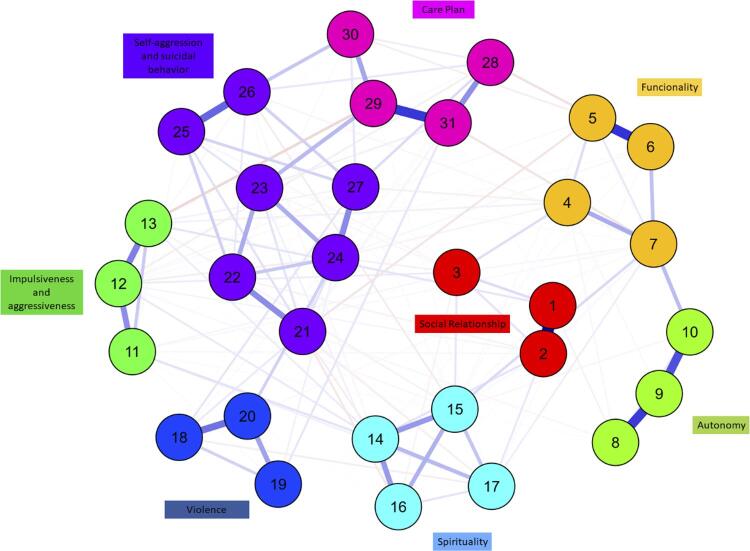
Network analysis.

Note: the numbers indicate the item that makes up the domain.

The items were positioned within the network close to their peers in the domains. Figure 2 shows that the items were appropriately associated with their peers, respecting the established domains, which indicates the sustainability and stability of the proposed model.

**Figure 3:**
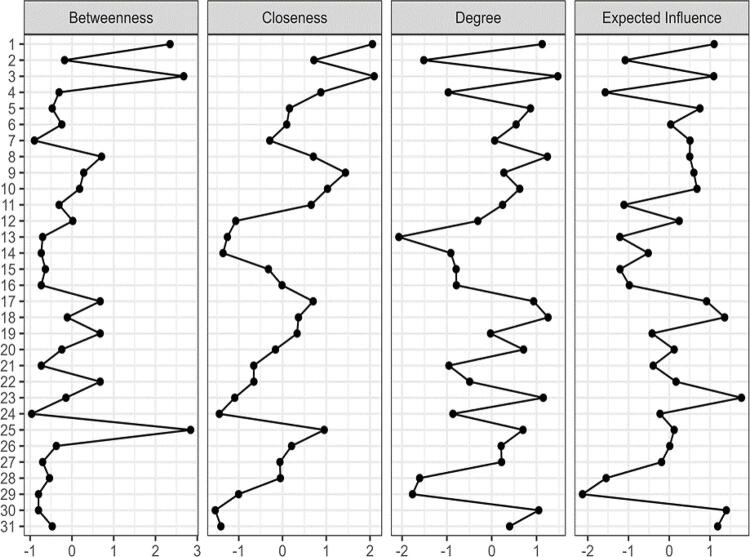
Centrality indices of standardized items (*z*).

For the standardized centrality indicators, the items (Figure 3) that presented the most relevant results were: for betweenness, items 1 “Do you have friends?”, 3 “Are you able to maintain friendships?”, and 25 “Does the user think about self-aggression?”. For closeness, items 1 “Do you have friends?”, 3 “Are you able to maintain friendships?”, and 9 “Do you perform your daily hygiene by yourself?”. For the strength/degree index, items 1 “Do you have friends?”, 3 “Are you able to maintain friendships?”, and 8 “Are you able to bathe by yourself?”, 18 “Has the user witnessed violence?”, 23 “Does the user plan suicide?”, and 30 “Is the user unaware of their disease?”, indicating that these items are those that have the strongest connection with the network. Items 1, 3, 23, 18, and 30 are also those with the greatest expected influence and, therefore, those with the greatest cumulative influence on the configuration of the model.

In short, both the CFA and network analysis results confirm a consistent and reliable model of the instrument, in accordance with the original model^9^obtained in the exploratory factor analysis.

## DISCUSSION

This study sought to present additional evidence of the CuidaSM’s internal structure, through CFA and network analysis. The results demonstrated that the primary indicators and model adequacy indices were established at satisfactory and consistent levels. Furthermore, the items were appropriately associated with their peers, respecting the established domains and confirming the stability of the proposed model.

In this sense, these findings endorse the contribution regarding the information obtained from the combination of techniques. The literature has described the need and contribution of tested models integrating multiple techniques, as this combination seeks to improve the precision and quality of instruments^11^. As Borsoom^25^points out, the use of network analysis or network psychometrics is part of a broader movement in psychology, which revolves around the analysis of human beings as complex systems, mainly because it allows the connection between components traditionally studied in isolation. Configuring itself as an emerging technique in psychometric studies.

This study, therefore, demonstrates additional validity evidence of CuidaSM, which presents eight dimensions distributed in 31 items, considering the user’s perception within the scope of their social relationships, functionality, autonomy, impulsiveness, aggressiveness and spirituality, in addition to counting on professional assessment regarding aspects related to violence, self-aggression, suicidal behavior and the care plan.

It is important to highlight that the development of the items was guided by a convergent understanding of the proposals of the psychosocial care model^5^, covering aspects that go beyond addressing signs and symptoms. In addition, it sought to incorporate specific questions about the care plan which, in more complex cases, can alert to the involvement of actions shared with other members of the multi-professional team.

It is also noted that, in the context of HCP, the CuidaSM scale is characterized as a promising instrument, as it provides support for the articulation between the points of the Psychosocial Care Network, as it can contribute to the management function—offering objective parameters that enable the preparation of health services programming for the different strata of care needs in MH—and with an objective communicational function, by enabling PHC services to use the same stratification parameter^2^.

In this way, according to the score established in the CuidaSM scale^9^, users can be classified as having low, medium, high or very high MHCN. It is expected that, based on these strata, services can establish parameters to organize care among those who can benefit from supported self-care technologies focused on PHC or those who require significant professional attention, which may even involve co-participation of specialized care.

It is noteworthy that, at an international level, discussions have been carried out about the organization and planning of MH services in environments with limited resources, especially through the stepped care model, in which individuals access treatments in a sequential, self-correcting manner^38^. Furthermore, recently, the adoption of a stratified approach has been incorporated into this debate^39^, strengthening the reflections triggered by the study.

The fact of adopting an intentional sample could represent a limitation for generalizing the results of this study. However, it is emphasized that numerous analyses and data processing were carried out to ensure that the instrument provided adequate, consistent evidence that legitimizes its application nationwide. Therefore, this study is distinguished by the fact that the analyses indicate that the items are valid for measuring the MHCN in federative units in Brazil’s five geographic regions.

Finally, it is recommended that future research investigate aspects of the implementation of the CuidaSM scale in the daily routine of PHC services and its applicability for decision-making in the planning of MH care, according to the needs identified in users.

## CONCLUSION

The study’s findings confirm a consistent and reliable model for CuidaSM, using a combination of techniques. Thus, considering the importance of using solid instruments in clinical practice, CuidaSM is as a promising tool for population-based management and network care organization, in line with the proposals of the HCP.
