# Supplementary material for: Brazilian Scale for Evaluation of Mental Health Care Needs: Additional Evidence
Source: Rev Saude Publica. 2024 Apr 1;57(Suppl 3):7s. doi: 10.11606/s1518-8787.2023057005347 (PMC11037904; doi:10.11606/s1518-8787.2023057005347)
Supplement: Translation

# Translation: Escala de Avaliação da Necessidade de Cuidado em Saúde Mental: evidências adicionais

**Authors:** Joana Moscoso Teixeira de Mendonça, Flavio Rebustini, Ana Alice Freire de Sousa, Ilana Eshriqui, Daiana Bonfim, Leticia Yamawaka de Almeida

**Affiliations:** I Brasil Hospital Israelita Albert Einstein. Centro de Estudos, Pesquisa e Prática em Atenção Primária à Saúde e Redes (CEPPAR). São Paulo, SP, Brasil; II Brasil Universidade de São Paulo. Escola de Artes, Ciências e Humanidades. Departamento de Gerontologia. São Paulo, SP, Brasil

**Keywords:** Saúde Mental, Atenção Primária à Saúde, Gestão da Saúde da População

## Abstract

**OBJETIVO:**

Investigar evidências de validade da Escala de Avaliação da Necessidade de Cuidado em Saúde Mental (CuidaSM).

**MÉTODOS:**

Trata-se de um estudo de natureza psicométrica, que busca evidências adicionais de estrutura interna. A coleta de dados foi realizada em 11 unidades básicas de saúde (UBS), que executam a metodologia da Planificação da Atenção à Saúde (PAS), distribuídas nas cinco regiões brasileiras. A versão preliminar da CuidaSM, contendo um bloco autorreferido pelo usuário e outro avaliado por profissionais da atenção primária à saúde (APS), foi aplicada em usuários com 18 anos ou mais que compareceram às UBS para consulta com profissional de nível superior. As técnicas de análise fatorial confirmatória e *network analysis* foram utilizadas para investigação das evidências de validade. Para os dados primários da análise fatorial confirmatória, utilizou-se as cargas fatoriais e o poder preditivo do item (R2). Foram adotados seis índices de ajustamento do modelo e a confiabilidade foi aferida por três indicadores por meio de *bayesian estimation*.

**RESULTADOS:**

Participaram do estudo 879 usuários. Pela análise fatorial confirmatória, as cargas fatoriais variaram de 0,43 a 0,99 e R2 de 0,19 a 0,98. Tanto os indicadores primários como os índices de adequação do modelo estabeleceram-se em níveis satisfatórios e consistentes. A *network analysis* apontou que os itens se associaram de forma adequada a seus pares, respeitando as dimensões estabelecidas, o que indica, novamente, a sustentabilidade e estabilidade do modelo proposto.

**CONCLUSÕES:**

Os achados do estudo confirmam um modelo consistente e confiável do instrumento, por meio da combinação de técnicas. Considerando a relevância do uso de instrumentos sólidos na prática clínica, a CuidaSM se apresenta como uma ferramenta promissora para a gestão de base populacional e organização do cuidado em rede, alinhada às propostas da PAS.

## INTRODUÇÃO

A Planificação da Atenção à Saúde (PAS) é uma metodologia que busca proporcionar, aos profissionais de saúde, o desenvolvimento de competências voltadas ao planejamento e organização da atenção à saúde, considerando as necessidades dos usuários em seu território. Em linhas gerais, a proposta visa a integração da Rede de Atenção à Saúde (RAS), utilizando estratégias de melhoria na organização dos macro e micro processos da atenção primária à saúde (APS) e da atenção ambulatorial especializada (AAE)^1^.

No contexto da APS, utiliza-se a metáfora da construção de uma casa para ilustrar o modelo teórico operacional da sua construção social, no qual os macro e micro processos básicos se constituem como alicerce e oferecem subsídios para o atendimento das demandas da população adscrita^1^. Nesta perspectiva, a estratificação de risco das condições crônicas, pautada no Modelo de Atenção às Condições Crônicas (MACC), emerge como elemento fundamental para o planejamento e a implementação das ações nos serviços, de acordo com a gestão de base populacional^1,2^.

Destaca-se que a realização deste processo, ao possibilitar a identificação das necessidades dos usuários e diferenciá-los por grupos/estratos, direciona a tomada de decisão do profissional em relação ao tipo de atenção, recursos e tecnologias específicos para o manejo clínico^2^ e organização da assistência em diferentes linhas de cuidado.

No âmbito da atenção em saúde mental (SM), diante de um cenário global de recursos escassos e da lacuna terapêutica para os transtornos mentais^3,4^, a APS tem se configurado como um componente estratégico para o cuidado^5,6^. Entretanto, o uso de ferramentas para quantificar e organizar esta demanda nos serviços tem sido considerado um desafio entre os profissionais^7^.

As linhas de cuidado em SM desenvolvidas pelo Ministério da Saúde^8^ recomendam o uso de instrumentos que, em geral, visam facilitar a detecção de condições específicas, como depressão, ansiedade e problemas relacionados ao uso de álcool. Contudo, nota-se a ausência de instrumentos que apoiem o profissional no escalonamento do cuidado em SM no território.

Neste sentido, a Escala de Avaliação da Necessidade de Cuidado em Saúde Mental (conhecida como CuidaSM)^9^ foi desenvolvida, alinhada ao conceito de gestão de base populacional, para facilitar o reconhecimento de necessidades específicas do cuidado em SM, configurando-se como ferramenta em potencial para colaborar com as equipes de APS na operacionalização do macroprocesso básico de estratificação e, por conseguinte, na provisão e organização do cuidado.

Estudo prévio demonstrou evidências de validade da CuidaSM, por meio de análise fatorial exploratória, desse modo, este instrumento é composto por 31 itens dicotômicos, contemplando cinco dimensões em um bloco autorreferido (Relações Sociais, Funcionalidade, Autonomia, Impulsividade e Agressividade, Espiritualidade) e três dimensões em um bloco aplicado por profissional de nível superior da APS (Violência, Autoagressão e Comportamento Suicida, Plano de Cuidados)^9^. A partir de sua aplicação, possibilita-se a classificação das pessoas usuárias da APS em quatro estratos de necessidade de cuidado em saúde mental (NCSM) (baixa, moderada, alta e altíssima)^9^.

Nesse contexto, a CuidaSM apoia o processo de escalonamento do cuidado em saúde mental a partir de dados objetivos sobre a NCSM. Além disso, apresenta o potencial de contribuir com a comunicação efetiva entre diferentes pontos de atenção da rede, com a tomada de decisão quanto a terapia certa, no momento certo, para a pessoa usuária certa, e, portanto, com a utilização mais racional dos recursos técnicos e humanos, auxiliando na programação do cuidado.

Considerando que até o momento não se encontram outros instrumentos validados para mensurar a NCSM na população brasileira, assim como a relevância de se utilizar, na prática clínica, instrumentos sólidos em termos de propriedade psicométrica e em consonância com as recomendações atuais acerca de múltiplas testagens para o ajuste de um instrumento^10^, este estudo objetivou investigar evidências de validade da CuidaSM.

## MÉTODOS

### Delineamento

Trata-se de um estudo de evidências adicionais de estrutura interna da CuidaSM, realizado por meio da análise fatorial confirmatória (AFC) e *network analysis*, segundo recomendação atual do American Educational Research Association, American Psychological Association e do National Council on Measurement in Education^10^. O estudo foi aprovado pelo Comitê de Ética em Pesquisa do Hospital Israelita Albert Einstein (CAAE: 12395919.0.0000.0071).

### Coleta de Dados

A versão preliminar da CuidaSM, composta por 43 itens (23 em um bloco autorreferido pelo usuário e 20 em bloco de avaliação clínica realizado por profissional de nível superior da APS), conforme descrita em estudo prévio^9^, foi aplicada em usuários cadastrados em 11 unidades básicas de saúde (UBS), totalizando 27 equipes de saúde da família. A coleta ocorreu entre novembro de 2021 a agosto de 2022, e foi conduzida por uma equipe de pesquisadores previamente treinados.

As UBS que compuseram o campo da pesquisa foram definidas com base na sua localização, em municípios que utilizam a metodologia da PAS e a inclusão de ao menos um serviço em cada região geográfica do país, sendo contempladas: uma UBS na região Norte (Roraima); uma UBS na região Nordeste (Pernambuco); duas UBS na região Centro-Oeste (Mato Grosso); cinco UBS na região Sudeste (sendo três em São Paulo e duas em Minas Gerais) e duas UBS na região Sul (Paraná).

Após o contato com a gestão dos serviços para pactuação do cronograma de atividades, o pesquisador responsável realizou encontros virtuais com as equipes das unidades para apresentação da proposta do estudo. Além disso, nesses encontros realizavam-se os esclarecimentos de dúvidas e preparação da coleta dos dados, que contava com os profissionais de saúde na avaliação do bloco clínico.

A operacionalização da coleta de dados nas UBS foi realizada em dois momentos. Inicialmente, os usuários eram abordados pelos pesquisadores nas salas de espera das UBS e convidados a participar da pesquisa, mediante leitura e manifestação de aceite do Termo de Consentimento Livre e Esclarecido. Neste momento, respondiam um questionário composto por itens sociodemográficos, perfil clínico e bloco autorreferido da escala CuidaSM.

Na sequência, durante a consulta do usuário com o profissional de saúde da APS, realizava-se a avaliação clínica da escala CuidaSM. Para este momento, utilizou-se uma versão impressa do instrumento que, ao final do dia, era recolhida pelos pesquisadores. O registro dos dados era realizado em um tablet, por meio da Research Electronic Data Capture (REDCap^©^).

Os critérios de elegibilidade para os usuários foram: ter 18 anos ou mais e estar na UBS para atendimento em consulta individual por médico, enfermeiro ou profissionais de saúde da equipe multidisciplinar. Foram excluídos aqueles que compareceram no serviço para atendimento de urgência, odontológico ou realização de procedimentos (como vacina, curativo e medicação) ou que não finalizaram o bloco de “avaliação do profissional de saúde”.

### Análise de Dados

#### Análise Fatorial Confirmatória (AFC)

Para os dados primários da AFC utilizou-se as cargas fatoriais e o poder preditivo do item (R^2^). Os índices de ajustamento do modelo adotados foram: χ^2^/df; Non-Normed Fit Index (NNFI > 0,95); Comparative Fit Index (CFI > 0,95); Goodness Fit Index (GFI > 0,95); Root Mean Square Error of Aproximation (RMSEA < 0,08) e Root Mean Square of Residuals (RMSR < 0,8). O modelo testado na AFC foi a solução fatorial encontrada no estudo^9^ inicial da análise fatorial exploratória.

A confiabilidade foi aferida por três indicadores: alfa de Cronbach^14^; Greatest Lower Bound (GLB)^15^ e Ômega^16^, os três por meio de *bayesian estimation*^17^.

## Network Analysis

Na última década houve uma extensão da aplicação da *network analysis* para diversos cenários e aplicações, tais como: avaliação de sintomas^18^, redes psicológicas e psicopatologias^19,20^, estresse pós-traumático^21,22^, esquizofrenia^23^, ansiedade^24^e desenvolvimento de instrumentos de medida^25^. Apesar disso, no Brasil são raros os trabalhos com *network analysis*^30,31^.

Cabe entender como ela pode ser útil na busca de evidências de validade. Segundo Newman^32^ (2010), a análise é composta por dois estágios: no primeiro, estima um modelo estatístico de dados, a partir do qual alguns parâmetros podem ser representados como uma rede ponderada entre as variáveis observadas; e no segundo estágio é analisada a estrutura da rede ponderada, usando medidas tiradas da teoria dos grafos.

Este estudo utilizou como estimador a técnica High-dimensional Undirected Graph Estimation (Huge)^33^e como critério o Extended Bayesian Information Criterio (Ebic). A Huge trabalha com dois procedimentos de estimação: 1) o algoritmo de busca em vizinhança^34^ e 2) o algoritmo gráfico de Lasso^35^. Os nodos dos gráficos foram posicionados utilizando o algoritmo de Fruchterman e Reingold^36^, que é baseado na força e conectividade entre os nodos. Cada nodo representa um item do instrumento.

Para avaliar o modelo gerado, foram adotados quatro indicadores: Intermediação (*betweeness*), que avalia a eficiência com que um nó se conecta aos outros; proximidade (*closeness*), que avalia a facilidade com que a informação atinge os outros nós a partir de um nó específico; força (*strength or degree*), que representa o quanto um nó está conectado com o resto da rede^37^; e, por fim, a influência esperada (expected influence), que visa avaliar a natureza e a força da influência cumulativa de um nó dentro da rede^23^ e, portanto, o papel que se pode esperar que ele desempenhe na ativação, persistência e remissão dela^38^.

Para ambas as técnicas foi aplicada uma *bootstrap* de 5.000. As análises foram efetuadas como JASP 16.04

## RESULTADOS

Aceitaram participar do estudo 1.219 pessoas, entre as quais 879 tiveram os dois blocos da CuidaSM preenchidos e foram incluídas neste estudo. Entre estas, a média de idade foi de 45 anos (DP = 16,7). A maioria era do sexo feminino (74,3%), casado ou em união estável (46,5%), não beneficiário de programas sociais (76,0%) e com nove anos ou mais de estudo (54,2%). Os participantes residiam majoritariamente na região Sudeste (58,4%), seguidas pelas regiões Sul (11,1%), Centro-Oeste (11,0%), Norte (5,4%) e Nordeste (4%).

Pela análise fatorial confirmatória, as cargas fatoriais variaram de 0,43 a 0,99, com erro padrão variando de 0.004 a 0.018 e a capacidade preditiva do item (R2) de 0,19 a 0,98 (Figura 1). Todos em níveis satisfatórios. Além dos indicadores primários, os índices de ajuste do modelo estabeleceram-se em: X2/df(245) = 1.64, p = 0,001; NNFI = 0,97, CFI = 0,98, GFI = 0,98, RMSEA = 0,06 e RMSR = 0,03.

**Figura 1:** Diagrama de caminhos.

Os índices de confiabilidade com estimação bayesiana foram de: alfa de Cronbach = 0.81 (IC95% 0,79–0,82), ômega de McDonald = 0,78 (IC95% 0,76–0,81) e GLB = 0.93 (IC95% 0.92–0,93). Todos os indicadores em nível adequados. Desta forma, tanto os indicadores primários como os índices de adequação do modelo estabeleceram-se em níveis satisfatórios e consistentes.

Os itens se posicionaram dentro da rede de forma próxima aos seus pares nos domínios. A Figura 2 demostra que os itens se associaram de forma adequada com seus pares, respeitando os domínios estabelecidos, o que indica a sustentabilidade e a estabilidade do modelo proposto.

**Figura 2:** Network analysis.

Nota: os números indicam o item que compõe o domínio.

Para os indicadores de centralidade normatizados, os itens (Figura 3) que apresentaram resultados mais relevantes foram: para a intermediação (*betweenness*), os itens 1 “Você tem amigos?”, 3 “Você consegue manter amizades?” e 25 “O usuário pensa em se agredir?”. Para a proximidade (*closeness*), os itens 1 “Você tem amigos?”, 3 “Você consegue manter amizades?” e 9 “Você realiza a sua higiene diária sozinho?”. Para o índice força/grau (*strenght/degree*), os itens 1 “Você tem amigos?”, 3 “Você consegue manter amizades?” e 8 “Você é capaz de tomar banho sozinho?”, 18 “O usuário foi testemunha de violência?”, 23 “O usuário tem planejamento suicida?” e 30 “O usuário desconhece a sua doença?”, indicando que esses itens são os que tem mais força de conexão com a rede. Os itens 1, 3, 23, 18 e 30 também são os com maior influência esperada e, portanto, os que apresentam a maior influência cumulativa para a configuração do modelo.

**Figura 3:** Índices de centralidade dos itens normatizados (*z*).

Em suma, tanto os resultados da AFC quanto da *network analysis* confirmam um modelo consistente e confiável do instrumento, conforme o modelo original^9^ obtido na análise fatorial exploratória.

## DISCUSSÃO

Este estudo buscou apresentar evidências adicionais de estrutura interna da CuidaSM, por meio de AFC e *network analysis*. Os resultados demonstraram que os indicadores primários e os índices de adequação do modelo estabeleceram-se em níveis satisfatórios e consistentes. Além disso, os itens se associaram de forma adequada aos seus pares, respeitando os domínios estabelecidos e confirmando a estabilidade do modelo proposto.

Neste sentido, estes achados endossam a contribuição quanto às informações obtidas da combinação entre técnicas. A literatura tem descrito a necessidade e a contribuição de modelos testados integrando múltiplas técnicas, uma vez que esta combinação busca melhorar a precisão e a qualidade dos instrumentos^11^. Como aponta Borsoom^25^, a utilização da *network analysis* ou psicometria de redes faz parte de um movimento mais amplo da psicologia, que gira em torno da análise dos seres humanos como sistemas complexos, principalmente por permitir a conexão entre componentes tradicionalmente estudados de forma isolada. Configurando-se como uma técnica emergente nos estudos de psicometria.

Este estudo, portanto, demonstra evidências de validade adicionais da CuidaSM, que apresenta oito dimensões distribuídas em 31 itens, considerando a percepção do usuário no âmbito de suas relações sociais, funcionalidade, autonomia, impulsividade, agressividade e espiritualidade, além de contar com a avaliação profissional frente aos aspectos relacionados à violência, à autoagressão, ao comportamento suicida e ao plano de cuidados.

É importante destacar que a elaboração dos itens foi norteada por uma compreensão convergente às propostas do modelo de atenção psicossocial^5^, contemplando aspectos que extrapolam a abordagem de sinais e sintomas. Além disso, buscou incorporar questões específicas acerca do plano de cuidado que, em casos mais complexos, podem alertar para o envolvimento de ações compartilhadas com outros membros da equipe multiprofissional.

Sinaliza-se ainda que, no contexto da PAS, a escala CuidaSM se caracteriza como um instrumento promissor, por fornecer subsídios para a articulação entre os pontos da Rede de Atenção Psicossocial (RAPS), uma vez que pode contribuir com a função gerencial – oferecendo parâmetros objetivos que possibilitam a elaboração da programação dos serviços de saúde para os diferentes estratos de necessidade de cuidado em SM – e com a função comunicacional objetiva, por possibilitar que serviços de saúde da APS utilizem o mesmo parâmetro de estratificação^2^.

Deste modo, conforme o escore estabelecido na escala CuidaSM^9^, os usuários poderão ser classificados em baixa, média, alta ou altíssima necessidade de cuidado em SM. Espera-se que, a partir desses estratos, os serviços possam estabelecer parâmetros para organizar o cuidado entre aqueles que podem se beneficiar de tecnologias de autocuidado apoiado e com foco na APS ou aqueles que exigem atenção profissional significativa, podendo envolver, inclusive, a coparticipação da atenção especializada.

Ressalta-se que, em nível internacional, as discussões acerca da organização e planejamento dos serviços de SM em ambientes com recursos limitados têm sido realizadas, especialmente, por meio do modelo de cuidado escalonado (ou *stepped care*), no qual os indivíduos acessam tratamentos de modo sequencial, autocorretivos^38^. Ademais, recentemente, a adoção de uma abordagem estratificada tem sido incorporada neste debate^39^, fortalecendo as reflexões disparadas pelo estudo.

O fato de ter adotado amostra intencional poderia representar uma limitação para generalização dos resultados deste estudo. No entanto, reforça-se que foram realizadas inúmeras análises e tratamento de dados para garantir que o instrumento se mostrasse com evidências adequadas, consistentes e que legitimam a sua aplicação em âmbito nacional. Desta forma, este estudo apresenta como diferencial o fato de as análises indicarem que os itens se mostram válidos para mensurar a necessidade de cuidado em saúde mental em unidades federativas das cinco regiões geográficas brasileiras.

Por fim, recomenda-se que pesquisas futuras investiguem aspectos da implementação da escala CuidaSM no cotidiano dos serviços de APS e sua aplicabilidade para a tomada de decisão no planejamento do cuidado em SM, de acordo com as necessidades identificadas nos usuários.

## CONCLUSÃO

Os achados do estudo confirmam um modelo consistente e confiável da CuidaSM, por meio da combinação de técnicas. Assim, considerando a relevância do uso de instrumentos sólidos na prática clínica, a CuidaSM se apresenta como uma ferramenta promissora para a gestão de base populacional e organização do cuidado em rede, alinhada às propostas da PAS.
